# Influence of temperature on the formation and encapsulation of gold nanoparticles using a temperature-sensitive template

**DOI:** 10.1016/j.dib.2015.09.035

**Published:** 2015-10-09

**Authors:** Noel Peter Bengzon Tan, Cheng Hao Lee, Pei Li

**Affiliations:** Department of Applied Biology and Chemical Technology, The Hong Kong Polytechnic University, Hung Hom, Kowloon, Hong Kong, PR China

**Keywords:** Gold nanoparticles, Core–shell microgel, Smart nanocomposites

## Abstract

This data article describes the synthesis of temperature-sensitive and amine-rich microgel particle as a dual reductant and template to generate smart gold/polymer nanocomposite particle. TEM images illustrate the influence of reaction temperature on the formation and in-site encapsulation of gold nanoparticles using the temperature-sensitive microgel template. Thermal stability of the resultant gold/polymer composite particles was also examined.

## 1 **Specifications table**

**Table:** 

Subject area	Materials sciences
More specific subject area	Smart metal/polymer nanocomposite particles
Type of data	Images (TEM) and schematic diagram on the synthesis of temperature-sensitive particle template (PNIPAm/PEI)
How data was acquired	TEM images of gold-loaded PNIPAm/PEI composite particles were observed using a transmission electron microscope (JEOL 100 CX II) at an accelerating voltage of 100 kV.
Data format	Analyzed
Experimental factors	Synthesis of core–shell microgel template (PNIPAm/PEI).
Experimental features	Effect of temperature on the formation and encapsulation of gold nanoparticles within the soft microgel (PNIPAm/PEI) template. Effect of thermal treatment of Au@PNIPAm/PEI composite particles
Data source location	Department of Applied Biology and Chemical Technology, The Hong Kong Polytechnic University, Hung Hom, Kowloon, Hong Kong, PR China.
Data accessibility	Data are available with this article.

## 2 Value of the data

• The one-pot synthetic approach of core–shell microgel particles and their resulting gold/polymer composite particles can easily be integrated to a scalable process in aqueous medium.
• The TEM images provided here can be used to establish comparability with other results and validate their data of using amine-rich microgel particle for gold nanoparticle formation.
• The effect of thermal treatment on the formation and encapsulation of gold nanoparticles using microgel template will be useful for other researchers who are developing smart catalysts, nanocomposites, and its biomedical applications.

## 3 Data

The data shown here provide support for our previous work on the facile synthesis of gold/polymer nanocomposite particles using polymeric amine-based particles [1]. The purposes of these data are: (1) providing detailed synthesis of temperature-sensitive microgel particles consisting of poly(*N*-isopropylacrylamide) cores and amine-rich polyethyleneimine shells (PNIPAm/PEI); (2) verifying the effect of reaction temperature on the formation and encapsulation of gold nanoparticles using the PNIPAm/PEI microgel particles as dual reductants and templates; and (3) evaluating thermal stability of resultant Au@PNIPAm/PEI composite particles.

## 4 Materials, methods and experimental design

### 4.1 Materials

Branched polyethyleneimine (PEI) (50% aqueous solution having a weight average molecular weight of 750,000), *N,N׳*-methylenebisacrylamide (MBA) and *tert*-butyl hydroperoxide (TBHP, 70% solution in water) were all purchased from Sigma Aldrich Chemical Co., and used without further purification. Spindle-crystals of *N*-isopropylacrylamide (NIPAm, Aldrich) were purified by a repeated recrystallization of the NIPAm monomer using a mixture of toluene and n-hexane (1:5 v/v). Hydrogen tetrachloroaurate (III) trihydrate (HAuCl_4_·3H_2_O) was purchased from Aldrich-Sigma and used as received. Deionized water or Milli-Q water was used for dilution and dispersion medium.

### 4.2 Synthesis of PNIPAm/PEI template

**Fig. 1:**
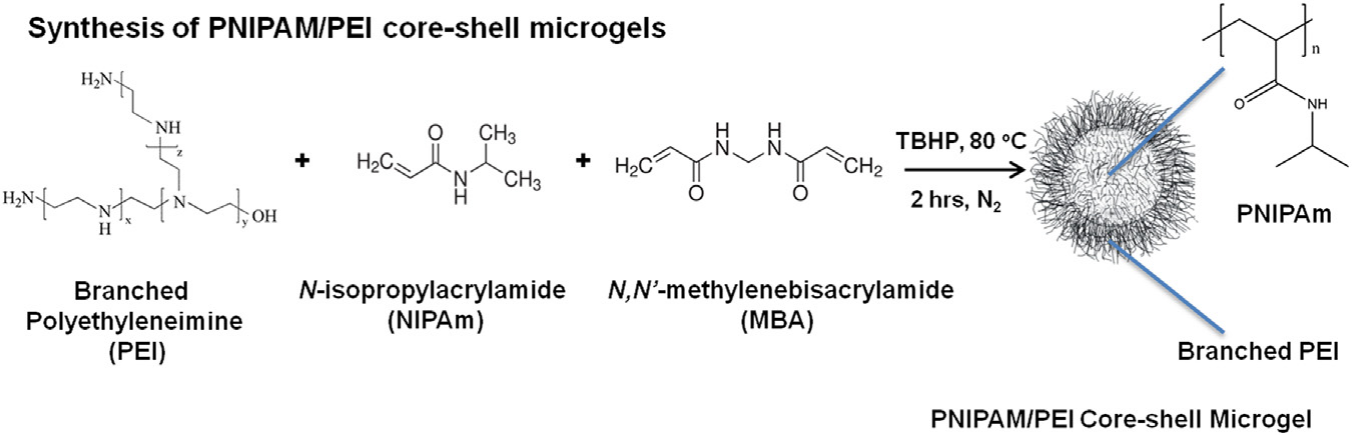
Schematic diagram on the synthesis of the soft core–shell microgels, poly (*N*- isopropylacrylamide)/polyethyleneimine (NIPAm to PEI weight ratio=4:1, 3% solids, pH 6.93 at 80 °C for 2 h). Average hydrodynamic diameter=348 nm, *zeta*-potential value of the PNIPAm/PEI microgel particles=34 mV).

Fig. 1 illustrates the synthesis of PNIPAm/PEI core–shell microgels according to our previously established procedure [2]. For a total weight of 107 g, PEI (1.27 g, 50% aqueous solution) was first dissolved in 50 mL water, followed by adjusting its pH to 7.0 with 2 M HCl solution. Purified NIPAm monomer (2.31 g) and MBA (0.23 g) were charged to the PEI solution and mixed in a three-necked water-jacketed flask equipped with a condenser, a magnetic stirrer, and an inlet tube. The mixture was purged with nitrogen for 30 min, followed by addition of TBHP solution (1 mM, 1.07 mL). It was continuously stirred at 80 °C for 2 h. The product of particle dispersion was subsequently purified by a repeated centrifugation at 18,000 rpm at 10 °C for 2 h. The purification cycle was repeated until conductivity of the supernatant was close to that of deionized water used.

### 4.3 Effect of temperature on the formation and encapsulation of gold nanoparticles in a soft microgel template

**Fig. 2:**
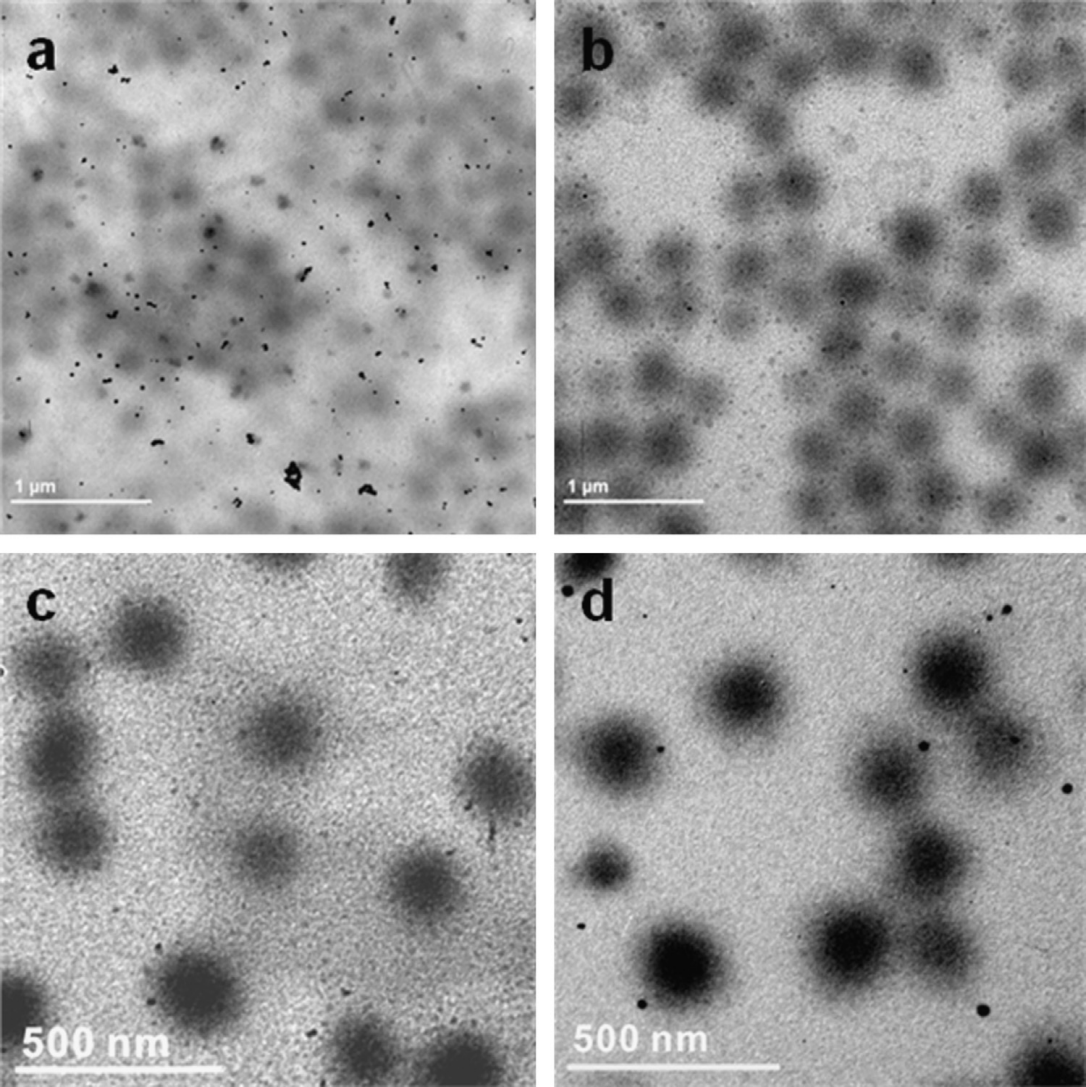
TEM images of AuNPs in PNIPAm/PEI templates synthesized under different solution temperatures: (a) 15 °C; (b) 25 °C; (c) 30 °C; and (d) 35 °C (reaction conditions: N/Au mole ratio=28.5. The reaction was stirred at 250 rpm for 2 h at pH 7.0).

Fig. 2 shows the effect of reaction temperature (at 5, 25, 30 and 35 °C) on the formation and encapsulation of gold nanoparticles using PNIPAm/PEI as a template. TEM images reveal that the best encapsulation performance of gold nanoparticles was between 25 and 30 °C, giving stable Au@PNIPAm/PEI composite particles with homogeneous distribution of gold nanoparticles around the particle shells. When the reaction was carried out at 15 °C, encapsulation ability of the microgel was obviously poorer than that at 25 °C since many gold nanoparticles located outside the templates. When reaction temperature was above the phase transition temperature of the PNIPAm, the temperature-sensitive polymer chains could contract, resulting in much less dense polymer shell. Thus, the entrapped gold nanoparticles could easily escape from the template.

### 4.4 Effect of thermal treatment on Au@PNIPAm/PEI composite particles

**Fig. 3:**
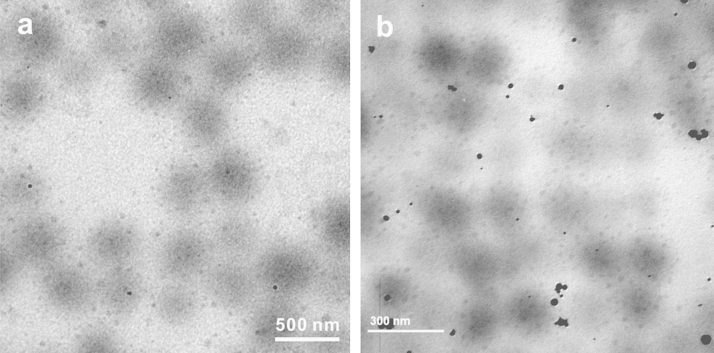
(a) AuNPs/PNIPAm/PEI composite microgels before thermal treatment; (b) after post-treatment of the composite particles at 92 °C for 1 h in aqueous (N/Au^3+^ molar ratio of 28).

Thermal stability of the resultant Au@PNIPAm/PEI composite particles was evaluated through treating the pre-formed composite particles at 92 °C for 1 h. TEM images as shown in Fig. 3 illustrate that majority of composite particles remained intact after the thermal treatment. The gold nanoparticles still appear blurry in the TEM images, indicating their coverage with the polymer. Only a few gold nanoparticles came out from the templates and formed aggregates. These aggregated gold nanoparticles appear to be much darker in the TEM image because they are not covered by the polymer.

## References

[1] Tan N.P.B., Lee C.H., Chen L., Ho K.M., Lu Y., Ballauff M., Li P. (2015). Polymer.

[2] Leung M.F., Zhu J., Harris F.W., Li P. (2004). Macromol. Rapid Commun..

